# Editorial: Police trauma, loss, and resilience

**DOI:** 10.3389/fpsyg.2022.1012970

**Published:** 2022-11-24

**Authors:** Konstantinos Papazoglou, Katy Kamkar, Peter Ian Collins, Michael David Schlosser

**Affiliations:** ^1^Pro Wellness Inc., Toronto, ON, Canada; ^2^The Power Project, San Diego, CA, United States; ^3^Department of Psychiatry, Temerty Faculty of Medicine, University of Toronto, Toronto, ON, Canada; ^4^Police Training Institute, University of Illinois at Urbana-Champaign, Champaign, IL, United States

**Keywords:** police resilience, police wellness, police stress, police trauma, loss

Prior research, practical experience, and clinical practice in the field of law enforcement support the fact that police work is challenging, stressful, and that exposure to potentially traumatic events is endemic to police work. Over the last couple of decades, research, policy, and clinical practice have also focused on police resilience and wellness. Exploring the impact, for instance, of gruesome crime scenes (e.g., decomposed dead bodies) on officers' health from a psychopathological perspective is one aspect of the phenomenon. On the other hand, many researchers and professionals have also placed emphasis on studying what makes police officers resilient and what strategies can help police officers maintain resilience and promote wellness. This is an intriguing topic allowing us to explore what helps officers thrive despite exposure to hundreds or even thousands of potentially traumatic events over the course of their career. In contrast, the average civilian would probably be exposed to a small number, if any, of potentially traumatic incidents over the course of their lives.

Considering current advances in research and clinical work with police we, as editors of present Research Topic, have advanced that a Frontiers Research Topic specifically focused on law enforcement trauma, loss, and resilience is imperative. To this end, we have envisioned the Research Topic as a forum that would open space for presentation of current research findings, clinical implications, policy recommendations, as well as a dialogue for future directions in this area of work. When we first established the current Research Topic, we were hopeful that scholars and practitioners in the area of law enforcement would embrace and endorse this initiative by submitting their articles for peer-review process and consideration for publication. Nevertheless, the final outcome exceeded our expectations. We can now proudly announce that the present Research Topic encompasses the work of many scholars and practitioners in the field who decided to submit their manuscripts for peer-review process and publication consideration in our Research Topic. This is further evidence of their appreciation of the importance of the topic as well as the potential of such topic in helping and guiding police researchers, professionals, and executives in the years to come in regards to promoting police resilience and wellness. The present Research Topic hosts the work of 95 authors from the United States, Canada, and other international countries, furthering the synergy of voices that, indeed, emanates from the 25 research articles published in our Research Topic.

The present Research Topic presents various topics under the overarching topic of police loss, trauma, and resilience. Topics such as trauma, loss, death in policing, moral injury, compassion fatigue, burnout, stress, resilience, and wellness are explored and discussed not only from an individual perspective but also from an organizational perspective. The collective role of the organization is also presented considering that resilience and wellness cannot flourish in the absence of organizational initiatives and support. Moreover, the manuscripts included in the present Research Topic vary in nature and may focus on theoretical conceptualizations, qualitative studies, as well as quantitative studies. What also makes this Research Topic unique and integral is that authors who contributed in this work come from different corners of the planet, which means that they convey, through their publications, their own knowledge and perspective about police loss, trauma, and resilience. That is, this Research Topic is transformed to an international platform of exchanging ideas, perspectives, appreciating values, and different cultural aspects in the context of law enforcement. Despite the international background of some of the authors, the main idea is that there are common denominators in policing across democratic countries. Therefore, when we consider the synergy of different voices and ideas we may then deliver some of those ideas to our own agencies and work as a way to promote research, policy, and clinical work.

To date, we have thousands of views and downloads from people from all over the world who are interested in studying the articles published in our Research Topic. We hope that this is only the beginning since exchange and sharing of knowledge is vital in helping scholars, policy makers and practitioners transfer advancements in the field to their own agencies in raising awareness and promoting resilience and wellness in their own organizations. As well, we anticipate that the present Research Topic will inspire and guide future researchers and practitioners to evolve their own ideas and, hence, develop and/or implement strategies in the topic of police trauma, loss, and resilience.

Lastly, we would like to express our gratitude to all the reviewers who meticulously and patiently reviewed all the manuscripts submitted for publication to the present Research Topic. Rigorous, accurate, and fair peer-review process is integral in promoting dissemination of scientific findings. As well, we would like to express our sincere gratitude and appreciation to the Frontiers team whom unequivocally dedicated their time and efforts to have this Research Topic achieve such great astonishing accomplishments of great quality of work which emanates from a large number of manuscripts. Enjoy reading and we strongly encourage you to disseminate the word about this unique collective scholarly work, police loss, trauma, and resilience.

## Author contributions

All authors listed have made a substantial, direct, and intellectual contribution to the work and approved it for publication.

## Conflict of interest

Author KP is the Founder and Director of ProWellness Inc. The remaining authors declare that the research was conducted in the absence of any commercial or financial relationships that could be construed as a potential conflict of interest.

## Publisher's note

All claims expressed in this article are solely those of the authors and do not necessarily represent those of their affiliated organizations, or those of the publisher, the editors and the reviewers. Any product that may be evaluated in this article, or claim that may be made by its manufacturer, is not guaranteed or endorsed by the publisher.

